# Changes in expression of *VGF*, *SPECC1L*, *HLA-DRA* and *RANBP3L* act with *APOE* E4 to alter risk for late onset Alzheimer’s disease

**DOI:** 10.1038/s41598-024-65010-7

**Published:** 2024-06-28

**Authors:** Sergio Branciamore, Grigoriy Gogoshin, Andrei S. Rodin, Amanda J. Myers

**Affiliations:** 1 Department of Computational and Quantitative Medicine, Beckman Research Institute of the City of Hope, Duarte, CA 91010 USA; 2 Department of Cell Biology, University of Miami Miller School of Medicine, Miami, FL 33136 USA; 3 Institute for Data Science and Computing, University of Miami Miller School of Medicine, Miami, FL 33136 USA; 4 Interdepartmental Program in Neuroscience, University of Miami Miller School of Medicine, Miami, FL 33136 USA; 5 Interdepartmental Program in Human Genetics and Genomics, University of Miami Miller School of Medicine, Miami, FL 33136 USA

**Keywords:** Late Onset Alzheimer's disease, Apolipoprotein E, APOE E4, Genetics, Gene expression, Bayesian networks, Computational models, Alzheimer's disease

## Abstract

While there are currently over 40 replicated genes with mapped risk alleles for Late Onset Alzheimer’s disease (LOAD), the *Apolipoprotein* E locus E4 haplotype is still the biggest driver of risk, with odds ratios for neuropathologically confirmed E44 carriers exceeding 30 (95% confidence interval 16.59–58.75). We sought to address whether the *APOE* E4 haplotype modifies expression globally through networks of expression to increase LOAD risk. We have used the Human Brainome data to build expression networks comparing *APOE* E4 carriers to non-carriers using scalable mixed-datatypes Bayesian network (BN) modeling. We have found that *VGF* had the greatest explanatory weight. High expression of *VGF* is a protective signal, even on the background of *APOE* E4 alleles. LOAD risk signals, considering an APOE background, include high levels of *SPECC1L*, *HLA-DRA* and *RANBP3L*. Our findings nominate several new transcripts, taking a combined approach to network building including known LOAD risk loci.

## Introduction

Apolipoprotein E (*APOE*) is a major risk factor in both early onset AD (EOAD, onset before 65) and late onset AD (LOAD, onset after 65)^1^. The APOE locus has three common haplotypes (*APOE* E2, *APOE* E3 and *APOE* E4) defined by two single nucleotide polymorphisms (rs7412 and rs429358). While the E4 haplotype is a major driver of risk^1^, the E2 haplotype appears to be protective^2^. We have found that considering *APOE* background can help to map additional factors involved in genetic risk^3^. These factors could be either acting in conjunction with *APOE* to increase or decrease risk or, conversely, could be acting independently of the *APOE* locus. It is through this context that we decided to map how the *APOE* E4 risk allele can affect ‘omics profiles.

Our prior work^4–7^ has involved analyzing expression profiles to determine the interplay of DNA, RNA, and protein in LOAD risk. We have used Bayesian network (BN) probabilistic / causal modeling to infer potential causality and we uncovered a potentially predictive transcript (in other words, a "hit") that replicated in two separate sets of data as well as cell culture models^5^. The choice of BN modeling (over alternative network methodologies, e.g., Markov networks and correlation-based co-expression networks) is motivated by a number of factors. First, the BNs ability (due to the BN directed acyclic graph structure) to filter out numerous spurious (indirect, or transitive) dependencies inherent to the multicollinear data rich in mutually dependent variables^8^, such as expression datasets. Second, the BNs ability to model non-additive higher-order relationships, both linear and non-linear. Third, the BNs ability to incorporate mixed data types (such as, in our case, expression data plus *APOE* haplotypes) into a unified analysis framework. Finally, and importantly, BN modeling is an explainable and segment-able statistical/machine learning activity, wherein each fragment of the network can be mechanistically and biologically interpreted as a probabilistic dependency and potential directional causality at high levels of granularity, and can be further scrutinized and in silico validated using “conventional” statistical tools. By concentrating on a variable of interest (*e.g.*, LOAD phenotype) and its immediate neighborhood in the network, we can perform a classification task with clearly defined local conditional probability tables, something that the dimensionality reduction / clustering / visualization methods, such as UMAP^9^ or t-SNE^10^, cannot directly address.

Here, we sought to extend our prior BN work using a few modifications. First, we included the *APOE* E4 risk haplotype in our analysis. Second, we prioritized data inputs using mutual information (MI) as a variable selection filter, as opposed to differential expression (DE). MI measures the informational gain for understanding the state of one variable given knowledge about the state of another variable. Rather than rely on statistically significant differences in means (DE), MI estimates the overall dependence between two variables. Mixed MI (MMI) can deal with both quantitative and qualitative variables in the same space. Additionally, relationships can be given ranks based on the MMI cumulative distribution function (CDF), with only inputs showing high levels of MMI with the variable of interest (here diagnosis of LOAD) included. The final difference from our prior work is that we have used a different platform for BN network modeling, BNOmics^11–13^. BNOmics uses a novel hybrid (constraint-based + search-and-score) heuristic algorithm to increase scalability by significantly shrinking the search space thus making a series of full BN modeling experiments with varying hyper-parameters (e.g., discretization schedules) computationally feasible (days instead of weeks) in our setting, increasing convergence and robustness. In addition, difficulties with handling both qualitative and quantitative measures in the same analysis (i.e., the mixed-variable problem) are solved by the novel MMI metric and by our use of adaptive maximum entropy-based discretization. This allows for robust handling of mixed type variables and their linear and non-linear interactions within a single analysis framework.

In summary, we used BNOmics to analyze our Human Brainome datasets^5^ including the *APOE* E4 risk haplotype in the model, as well as performed separate analyses in the group of individuals who were *APOE* E4 positive (Group 1) and *APOE* E4 negative (Group 2). Input data was selected by the transcripts which best explained LOAD status (scored as presence (1) / absence (0) of pathologically confirmed LOAD), and expression profiles were discretized to maximize both biological interpretability and rigor and reproducibility (by insuring convergence in the search space). We were focused on searching for de novo hits that were protective against *APOE* E4 risk as well as those that contributed to *APOE* E4 risk.

### Subjects and methods

#### Human brain tissue samples

Sample sets are from The Human Brainome series^5^. Our KRONOSII series was obtained from 21 National Alzheimer’s Coordinating Center (NACC) brain banks and from the Miami Brain Bank as previously described^4,7,14^. Additional cohorts were obtained in the same manner as the original US series. Our criteria for inclusion were as follows: self-defined ethnicity of European descent (in an attempt to control for the known allele frequency differences between ethnic groups), neuropathologically confirmed AD or no neuropathology present, and age of death greater than 65. Neuropathological diagnosis was defined by board-certified neuropathologists according to the standard NACC protocols^15^. Samples derived from subjects with a clinical history of stroke, cerebrovascular disease, Lewy bodies, or comorbidity with any other known neurological disease were excluded. Alzheimer’s disease or control neuropathology was confirmed by plaque and tangle assessment with 45% of the entire series undergoing Braak staging^16^.

The RUSH series includes deceased subjects from two large, prospectively followed cohorts maintained by investigators at Rush University Medical Center in Chicago, IL: The Religious Orders Study (ROS) and the Memory and Aging Project (MAP). The ROS cohort, established in 1994, consists of Catholic priests, nuns, and brothers from 40 groups in 12 states who were at least 55 years of age and free of known dementia at the time of enrollment. The MAP cohort, established in 1997, consists of men and women primarily from retirement facilities in the Chicago area who were at least 53 years of age and free of known dementia at the time of enrollment. All participants in ROS and MAP sign an informed consent agreeing to annual detailed clinical evaluations and cognitive tests, and the rate of follow-up exceeds 90%. Similarly, participants in both cohorts signed an Anatomical Gift Act donating their brains at the time of death. The overall autopsy rate exceeds 85%. The ROS and MAP cohorts were analyzed jointly since they were designed to be combined, are maintained by a single investigative team, and a large set of phenotypes collected are identical in both studies^17^. More detailed information regarding the two cohorts can be found in previously published literature^18^.

The series included 475 samples in the KRONOSII set (257 controls, 218 cases; age range 65–105; 58% female) and 306 samples in the replicate set (162 controls, 144 cases; age range 66–104; 63% female). Samples were chosen by the diagnostic criteria as stated above as well as the availability of high quality RNA. All samples were assessed for ancestry using principle components analysis and the 1000G dataset^19^. Samples in our study cluster with European ancestry (CEU, FIN, GBR, IBS populations).

#### Ethics statement

The research for this project fails under 45 CFR 46.101(b)(4). Human Subjects exemption #4 states research involving the collection or study of existing data, documents, records, pathological specimens, or diagnostic specimens, if these sources are publicly available or if the information is recorded by the investigator in such a manner that subjects cannot be identified, directly or through identifiers linked to the subjects. In both series, samples were de-identified before receipt, and the study met human studies institutional review board and HIPPA regulations. Consents for biobanking are obtained from the source brain banks. This work is declared not human-subjects research and is IRB exempt under regulation 45 CFR 46. See the Acknowledgements section for a list of individual sites that contributed samples to this effort.

#### Data collection

Sample data was adjusted for several biological covariates (gender, age at death and cortical region) and several methodological covariates (institute source of sample, post-mortem interval, detection and hybridization date). Adjustment methodologies were as in the prior report^5^.

#### Data analysis-networks

**Figure 1:**
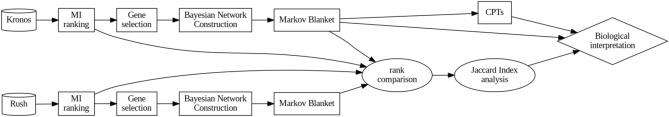
Pipeline. Shown is the overview of all computational procedures performed in the KRONOSII and RUSH datasets. As mentioned in the text, the matrix of gene expression was reduced to n = 2000 using MI for each dataset independently prior to BN construction. Markov Blankets (MBs) were captured from the full BN structures to determine top hits. Reproducibility between outputs was determined by rank comparison of MB hits and reproducibility of inputs was determined by the JI. Conditional Probability Trees (CPTs) were only built for KRONOSII given the lack of reproducibility between sets with respect to APOE genotype and the fact that KRONOSII gave the most robust results. The CPTs along with the BNs provide biological interpretability since we have the order to the network structure from the BNs and risk/resilience/expression pattern x genotype relationships from the CPTs.

Our focus was on the construction of high-dimensional multimodal Bayesian networks (BNs). As before^5^, we analyzed the KRONOSII and the RUSH datasets separately. Our analysis pipeline is shown in Fig. 1. *APOE* status was defined as either having no *APOE* E4 alleles (*APOE* negative) or at least one *APOE* E4 allele (*APOE* positive).

All analyses were performed using the BNOmics platform. BNOmics is a highly scalable open-source universal-purpose BN modeling and visualization software developed by us previously^11,12,20^. Each analysis involved the following:

*MMI.* The goal of the MMI-based variable selection/ranking was to select a more compact set of transcripts for subsequent BN analysis. We selected two series of 2000 top expression transcripts from the KRONOSII and RUSH data, respectively, as 2000 constituted a reasonable compromise between the computational efficiency of the full BN construction (hours to 1–2 days for each BN) and low probability of false negatives (given the smooth and monotonic nature of the MMI curves and the fact that the resulting Markov blankets of LOAD phenotype contained no more than 10–30 variables).

*DISCRETIZATION.* In order for our BN analyses to proceed, expression data first must be discretized. We used the multinomial model with the maximum entropy-based discretization, as the linear Gaussian model is not always a good fit with the transcript expression data^21^. We discretized the continuous transcript expression data into 3 bins (“high-medium–low”), which showed stable BN convergence and was also the most convenient discretization schedule from the biological interpretation standpoint. To insure that our discretization was robust and did not create artifacts such as false dependencies or independencies, we additionally plotted the full distribution using the ggstatsplot package in R^22^ for each of our main targets (continuous values) against disease risk.

*BNOmics.* We used a hybrid (constraint-based + search-and-score) algorithm with MDL scoring function previously described in^11,12,20^, with 20 restarts. BN edges are directed; however, a directed edge in the BN does not necessarily imply either directional causality or the biological hierarchy. Rather, edge directionalities describe the dependency structure between the variables (the factorization of the joint probability of the variables in the BN) that is most likely given the data, largely resolving multicollinearity issues. To identify the most salient transcripts, Markov neighborhoods (MNs) of the primary variable of interest (LOAD status, denoted as “DX” node in the networks) were used. MN is a simplification of the more rigorous concept of the Markov blanket (MB) that includes only the variables that are immediately adjacent to the variable/node of interest (such as DX) in the network. MB, consisting of the node’s “parents”, “children”, and other “parents” of the node’s “children”, is a superset of MN; given the MB of the variable of interest, it is independent of the remaining variables in the network, allowing us to concentrate on a small subset of variables to identify and highlight potential hits.

#### Data analysis-dataset similarity

To examine global similarity in transcript profiles, for each dataset (D), KRONOSII (K) and RUSH (R), we computed the Mixed Mutual Information (MMI)^13,23^ between the level of expression of each transcript *i (*${g}_{i}$*)* and the corresponding binary variable associated with the LOAD status (DX) as $MM{I}^{D}\left({g}_{i},DX\right)$. To simplify notation, we define ${I}_{i}^{D}=MM{I}^{D}\left({g}_{i},DX\right)$ . Subsequently, we define two transcript subsets r and k as ${r}_{p}=\left\{{g}_{i} : MM{I}_{i}^{R}>{x}_{R}\right\}$, and ${k}_{p}=\left\{{g}_{i} : MM{I}_{i}^{K}>{x}_{K}\right\}$ , with ${x}_{D}$ corresponding to the $p$ percentile value in the $MM{I}^{D}$ distribution. We used the Jaccard Index (JI) to measure the similarity between ${r}_{p}$, ${k}_{p}$. JI is defined as the size of the intersection divided by the size of the union of the sample sets. In our analysis we have computed JI using different cut-offs of MMI as $JI\left(G,p\right)=\frac{{r}_{p}\cap {k}_{p}}{{r}_{p}\cup {k}_{p}}$, with $G$ being the set of all transcript labels in KRONOSII and RUSH. Similar to a common practice in transcript-set enrichment analysis, a hypergeometric distribution was used to compute the probability (${P}_{val}$) of observing two subsets sampled randomly without replacement from $G$ of size equal to ${r}_{p}$ and ${k}_{p}$ and with JI greater than or equal to that observed for the corresponding ${r}_{p}$ and ${k}_{p}$. *APOE* genetic similarity between the two datasets was determined by looking at haplotype frequencies.

#### Data analysis-visualizations

MBs of the variable of interest (LOAD status / DX) were visualized as parts of the full BNs. Notably, these are the fragments of the full BNs, and not the small BNs built from the further reduced variable sets. The MBs included dependencies between the peripheral variables. The MMI CDFs were plotted for KRONOSII and RUSH, and specific hits were mapped to determine how much explanatory weight they had in each dataset according to a univariate MMI criterion. To dissect the effects (and their directions) of top BN-identified hits, two-level Conditional Probability Trees (CPTs) were created using the local conditional probability tables. CPT visualizations are intuitively similar to the conventional decision trees.

## Results

**Figure 2:**
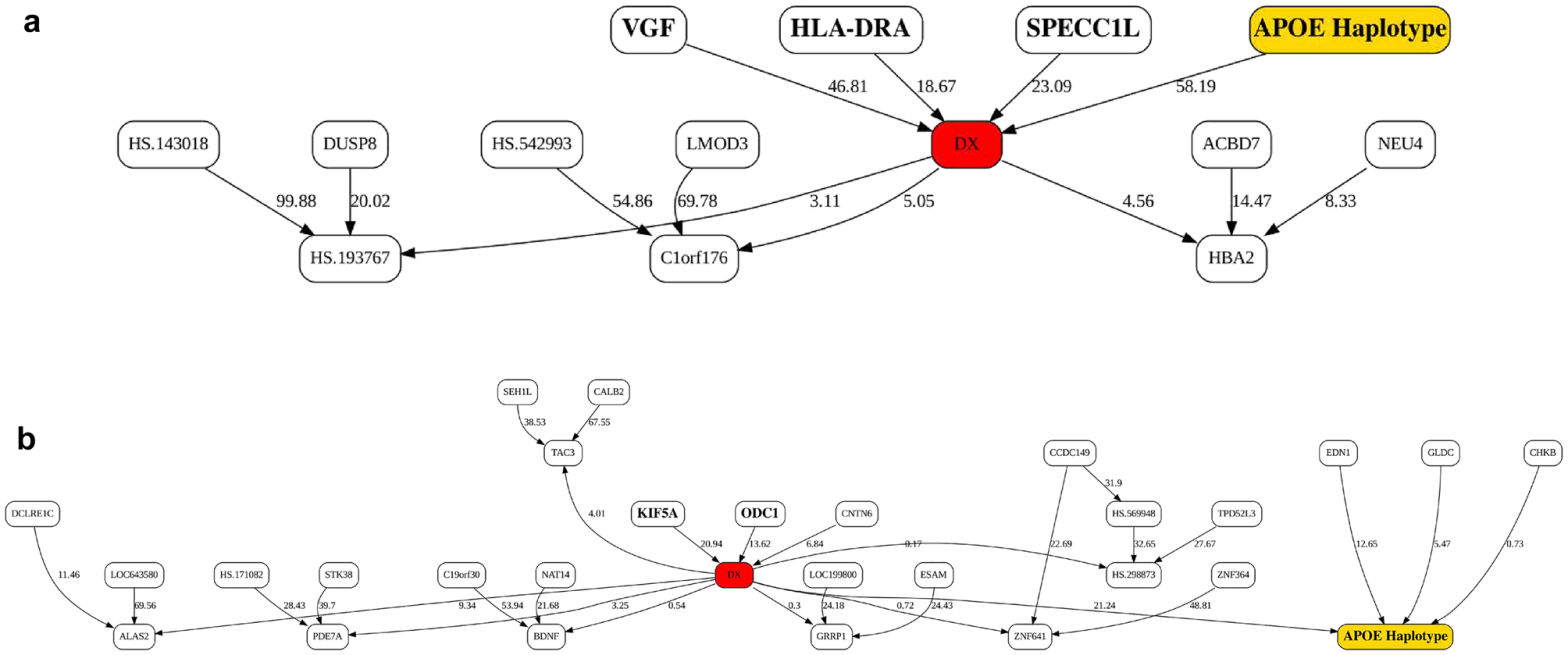
Markov Blankets (MBs) of the LOAD status in the full Bayesian Networks (BNs). Shown are the full MBs for (**A**) KRONOSII MB, top. (**B**) RUSH MB, bottom. “Bolded” nodes highlight the top hits. Numbers are the explanatory weights (edge strengths) and are proportional to the marginal likelihood ratio of the scoring functions of the model with the edge to the model without the edge, given the data. Edge strengths are unbound but can be compared to each other within a BN, with APOE Haplotype (gold node) serving as an expected positive control. DX (red node) is LOAD status scored as presence or absence of LOAD. The MB of DX is the set of variables in BN such that DX is conditionally independent of all the other variables given the variables in the MB (see text for details). Edge directionalities in the BN describe the dependency structure between the variables, i.e., the factorization of the joint probability of the variables in the BN, that is most likely given the data. Edge directionalities do not necessarily imply either directional causality or biological hierarchy. Top novel hits were identified as the highest-scoring nodes in the immediate Markov neighborhood (MN) of DX that were not APOE Haplotype.

Figure 2 depicts the MB of LOAD diagnosis (DX) for the 3-bin discretized expression data plus *APOE* genotype for KRONOSII and RUSH. We obtained converging structures in both sets. The top hits in KRONOSII were *APOE* E4 presence/absence, VGF Nerve Growth Factor Inducible (*VGF*), Sperm Antigen with Calponin Homology and Coiled-Coil Domains 1 Like (*SPECC1L*), and Major Histocompatibility Complex, Class II, DR Alpha (*HLA-DRA*). The top hits in RUSH were *APOE,* Kinesin Family Member 5A (*KIF5A*) and Ornithine Decarboxylase (*ODC1*). Violin plots of normalized, but not discretized profiles for all top targets are given in Supplementary Fig. 1 demonstrating our MB results (dependencies) were not due to possible discretization artifacts. Of note, for the small set of top explanatory hits for LOAD, only *APOE* E4 presence/absence replicated between the two datasets.

To investigate why there was little replication, we compared both the expression input from both KRONOSII and RUSH as well as examined the haplotype frequencies for *APOE* E4. We used the JI to compare the MMI expression datasets and found that at high levels of mutual information (> 60%), as we had in our top 2000 MMI input sets, the KRONOSII and RUSH expression datasets overlap, as measured by the JI (red line, Supplementary Fig. 2A). This similarity is statistically more significant than a random distribution of the JI (Supplementary Fig. 2B). From this we conclude that as prior^5^, the KRONOSII and RUSH expression datasets on their own are globally similar.

In this study, we are interested in including genetics in our modeling, specifically the *APOE* haplotype. To examine whether this was a factor impeding reproducibility, we plotted the frequencies of the main *APOE* haplotypes in each dataset. Of note, in Supplementary Fig. 3 there is a very low number of *APOE* E4s in the RUSH cohort. This effectively reduces the cohort size contributing to mapping effects by ~ 40% (KRONOSII *APOE* E4 positive controls n = 56; RUSH *APOE* E4 positive controls n = 22; KRONOSII *APOE* E4 positive cases n = 150; RUSH *APOE* E4 positive cases n = 57). This difference in *APOE* haplotype frequencies was not just due to sampling, given the KRONOSII set is larger (n = 475) than the RUSH dataset (n = 306). Examining APOE frequencies in additional RUSH samples (n = 458) where expression was not profiled due to tissue availability and/or quality, the *APOE* haplotype frequencies were very similar to the current RUSH set (*APOE* E22 current set = 1%; expanded set = 0.9%; *APOE* E23 current set = 13.6%; expanded set = 12.9%; *APOE* E33 current set = 58.5%; expanded set = 59.8%; *APOE* E24 current set = 2.7%; expanded set = 2.6%; *APOE* E34 current set = 23.1%; expanded set = 22.1%; *APOE* E44 current set = 1%; expanded set = 1.3%).

This reduction in power can clearly be seen in Fig. 2, where the explanatory weights (edge strengths) for diagnosis and APOE in RUSH are ~ 40% of those in KRONOSII (RUSH *APOE* E4 explanatory weight = 21.24; KRONOSII *APOE* E4 explanatory weight = 58.19). While proportionality of the BN scoring function (MDL, in our case) to the sample size makes direct inter-dataset comparisons and transfer learning between the datasets (cohorts) difficult, we believe it is likely this reduction in power due to less E44 haplotypes in the RUSH cohort is contributing to the lack of reproducibility; generally, we are seeing much larger effects in KRONOSII. We therefore decided to focus on the KRONOSII dataset for uncovering effects. We discuss the limitations of network comparisons in the DISCUSSION section below.

**Figure 3:**
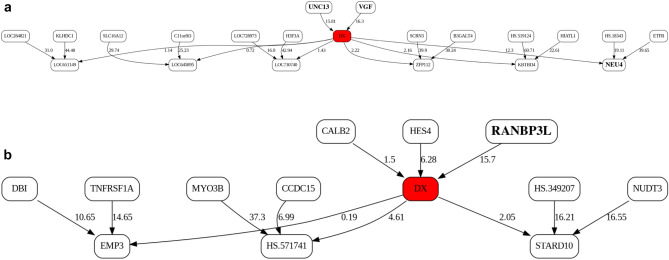
Markov Blankets (MBs) of the LOAD status in the full Bayesian Networks (BNs) in *APOE* E4 negative and *APOE* E4 positive samples, KRONOSII dataset. A. MB from individuals without *APOE* E4 haplotypes, top. **B** MB from individuals with APOE E4 haplotypes, bottom. See Fig. 2 legend for details. As prior, "bold" indicates top hits (UNC13, VGF, NEU4, RANBP3L).

To attempt to validate the results in KRONOSII, we split the dataset into *APOE* E4 positive individuals and *APOE* E4 negative individuals and ran these two groups separately. While not as ideal as a completely independent dataset, this approach does serve as a validation of effects given that MMI predictions and BN structures are produced independently from the prior runs using the entire KRONOSII series. Figure 3 shows the MB for the two separate groups of *APOE* E4 positive and *APOE* E4 negative samples in KRONOSII. Again, *VGF* is a top hit. In the KRONOSII group without the *APOE* risk haplotype, *VGF* and Homo sapiens unc-13 homolog B (*UNC13B*) had the greatest explanatory weight for pathologically confirmed LOAD. In the KRONOSII group with the *APOE* risk haplotype, RAN Binding Protein 3 like (*RANBP3L*) had the greatest explanatory weight for pathologically confirmed LOAD. Violin plots of normalized, but not discretized profiles for all top targets are given in Supplementary Fig. 4.

**Figure 4:**
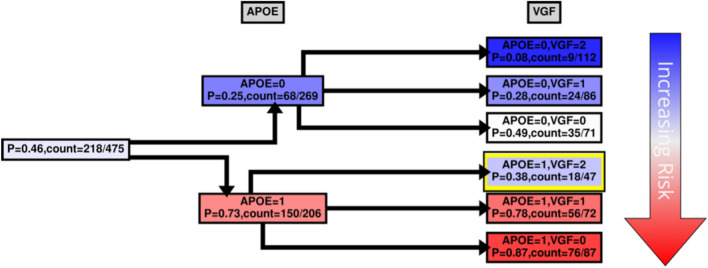
*VGF* CPT. Shown in Figs. 4, 5, 6 and 7 and Supplementary Figs. 3, 4 are the conditional probability trees (CPTs) for each hit (here, in Fig. 4, VGF). The goal of the CPT is to visualize expression direction on the background of genetic relationships. LOAD risk is associated with the absence (0) or presence (1) of specific *APOE* haplotypes and varying levels of VGF expression. The VGF expression level was divided into three categories based on the maximum entropy of the VGF expression distribution: low (0), medium (1), and high (2). The marginal probability of LOAD in the KRONOSII dataset, P(DX = 1), and the corresponding counts are shown at the CPT’s “root” (1^st^ column from the left). The conditional probability given APOE is shown in the first tree layer (2^nd^ column from the left): P(DX = 1|APOE = x) with x = 0,1. The conditional probability given APOE and VGF P(DX = 1|APOE = x, VGF = y) with y = 0, 1, 2 is shown in the second tree layer (3^rd^ column from the left). The color scheme (blue – white – red) reflects an increasing risk of LOAD (vertical arrow on the right); correspondingly, the second tree layer is ordered by increasing LOAD risk. The yellow box highlights the protective effect of high VGF expression on the background of having the *APOE* E4 haplotype.

**Figure 5:**
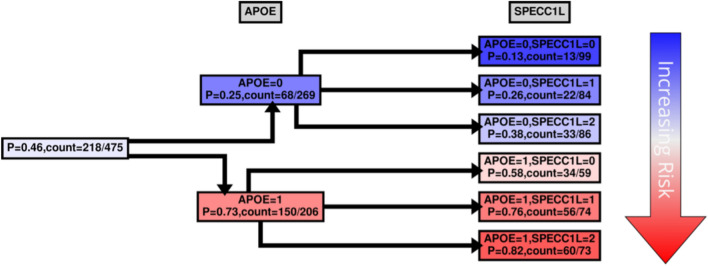
*SPECC1L* CPT. Same as in Fig. 4 with the exception that in the second tree layer (3^rd^ column from the left) the probability is computed for *SPECC1L*.

**Figure 6:**
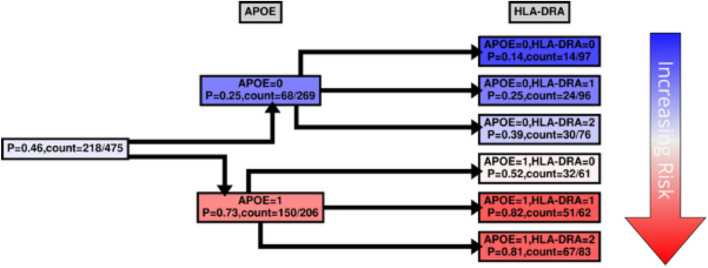
*HLA-DRA* CPT. Same as in Fig. 4 with the exception that in the second tree layer (3^rd^ column from the left) the probability is computed for *HLA-DRA*.

**Figure 7:**
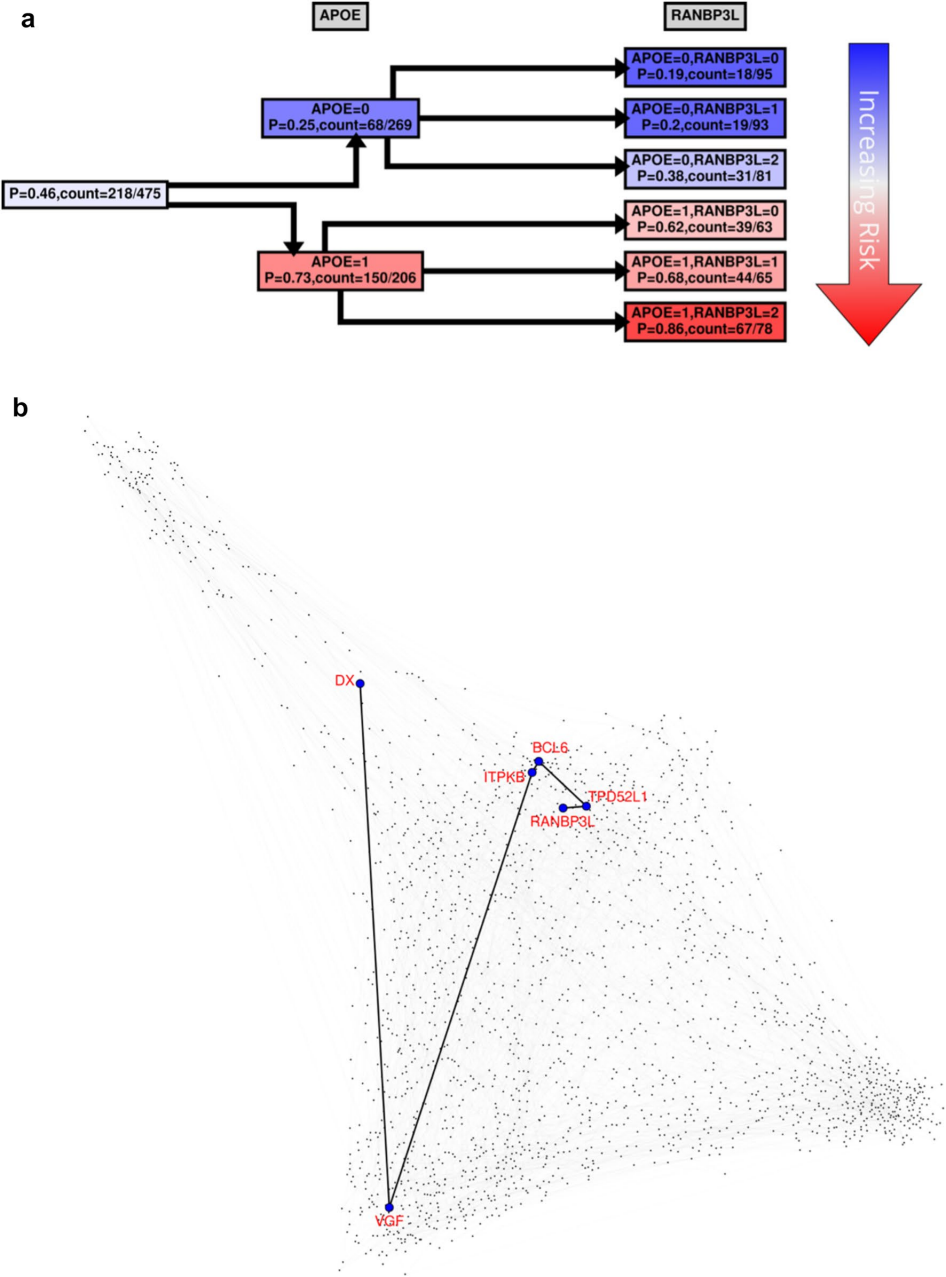
(**A**) *RANBP3L* CPT. Same as in Fig. 4 with the exception that in the second tree layer (3^rd^ column from the left) the probability is computed for *RANBP3L*. (**B**) *RANBP3L* dependency path in the full Bayesian Network (BN) for SET1. Full BN comprising top 2000 most informative (as measured by MMI) genes (represented by dots) is shown. The undirected dependency path between *RANBP3L* and DX (LOAD status) is highlighted. (Note that in this representation the edge lengths do not correspond to any meaningful measures and are merely for visual clarity).

While MBs indicate patterns of dependencies and strengths, they do not indicate how the dependency is manifested, *e.g.*, whether the transcript is highly expressed with *APOE* E4 haplotypes, or vice versa. We were interested to see how our case and control groups split by the level of expression and copies of *APOE* E4 haplotype. Figures 4, 5, 6 and 7 and Supplementary Figs. 5, 6 show conditional probability trees (CPT), which are decision tree-like visualizations of the conditional probability tables for each of the main hits. We found that *VGF*, *SPECC1L*, *HLA-DR* and *RANBL3L* gave strong consistent signals and acted in concert with *APOE* E4 haplotypes. While *UNC13* and *NEU4* gave strong signals in the network, there was less of a consistent (in its direction) signal.

High levels of *VGF* and no *APOE* alleles were most commonly found in the group of controls, with only 8% of the samples in this set having a pathologically confirmed diagnosis of LOAD (Fig. 4 top box, *APOE* = 0, *VGF* = 2). The greatest proportion of cases was found in the group of individuals with *APOE* 4 haplotypes and low levels of *VGF* (Fig. 4 bottom box, *APOE* = 1, *VGF* = 0). Of interest, there is a risk reduction in individuals with high levels of *VGF*, *even if they possess APOE E4 alleles* (Fig. 4 yellow highlight, *APOE* = 1, *VGF* = 2), indicating that *high levels of VGF can be protective on an APOE background*. This is a significant effect (comparing high *VGF* expression and *APOE* E4 presence with low *VGF* expression and *APOE* E4 presence OR = 2.2810; 95% CI 1.2217–4.2588, p-value = 0.0096) with an effective reduction in probability of ~ 40%.

For risk effects, we have mapped *SPECC1L*, *HLA-DRA* and *RANBP3L*. All of these elevate risk with increasing expression in a linear fashion with the *APOE* E4 haplotype. *SPECC1L* (Fig. 5) and *HLA-DRA* (Fig. 6) were seen without splitting the KRONOSII cohort by *APOE* E4 haplotypes (see Fig. 2). *RANBP3L* (Fig. 7A) was only ranked in the network when we split the cohort by *APOE* E4 haplotype (see Fig. 3). To investigate this effect, we traced the path of *RANBP3L* in the full cohort (Fig. 7B). *RANBP3L* was "missed" in the full dataset because it is steps removed from the diagnosis (DX) signal and in terms of network informative content it acts through *VGF*. *VGF* is our strongest signal, and thus, through splitting the sample into *APOE* E4 negatives, where *VGF* has the most effect given it is protective, and *APOE* E4 positives, where *VGF* has less of an effect, we are able to uncover *RANBP3L*.

As stated previously, *UNC13* (Supplementary Fig. 5) and *NEU4* (Supplementary Fig. 6) did not give a consistent signal in the CPT analysis. For *UNC13* consistency in expression is only seen in the *APOE* E4 haplotype absent set (APOE = 0 branch, top of figure). Note that in multinomial (and therefore multi-bin) BN analysis with more than two bins, relationships do not have to be linear; therefore, “consistency” in this sense indicates relationships with sustained “low-medium–high" or “high-medium–low" directions. There is a modest increase in the probability of LOAD with high *UNC13* expression if there are no *APOE* E4 haplotypes present. This is logical given these hits were only uncovered in the split analysis under the condition where the input KRONOSII dataset had no *APOE* E4 alleles (Fig. 3A). *NEU4* was also found in the MB of the network built from individuals without *APOE* E4 haplotypes; however, unlike *UNC13* there was no real relationship between high levels of expression or low levels of expression and risk. This suggests that, first, the minimum description length (MDL) scoring function in BNs tends towards higher sensitivity in general and, second, multinomial models capture differences at the distribution level that might not directly translate into the sustained (linear) directions. Therefore, “borderline” (lower) edge strengths in the MBs should be further scrutinized using the local probability tables (visualized as CPTs) and validated using traditional statistical univariate tests.

**Figure 8:**
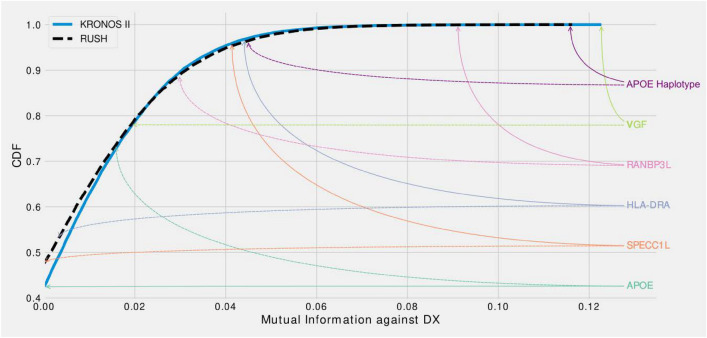
Cumulative Distribution Function (CDF) of the MMI. For each variable in the dataset with respect to DX the CDF was calculated from the MMI data. KRONOSII in solid blue; RUSH in dashed black. Rankings of selected genes are reported for KRONOSII (solid arrows) and RUSH (dashed arrows). APOE and APOE_Genetic represent the level of expression and haplotype state, respectively. The higher a hit is on the graph, the more explanatory weight there is for the state of diagnosis (i.e. whether or not samples have LOAD). As can be seen, the largest MMI signal was *VGF* in the KRONOSII dataset, followed by APOE Haplotype in the KRONOSII dataset. In RUSH, the largest signal was with APOE Haplotype, even though there were not as many *APOE* E4 samples as in KRONOSII. The next highest hit in RUSH was *RANBP3L*, which was also found in KRONOSII when we performed the analysis in the set of *APOE* E4 positive individuals. In both datasets, APOE gene expression (turquoise solid and dashed lines) was not an explanatory factor.

Finally, we were interested to search for the main effects we found in KRONOSII in RUSH and compare the level of MMI (univariate metric that does not account for multicollinearity and does not distinguish between direct and transitive dependencies) with diagnosis for each transcript. We considered the RUSH cohort as well, because while the reduced number of *APOE* E4 samples inhibits the power to map *APOE* E4 contingent structures, we still may be able to map effects considering MMI, which is a direct relationship between the hits and LOAD diagnosis. Figure 8 graphs the MMI CDFs for KRONOSII and RUSH, considering our main hits. As can be seen, *VGF* and *APOE* E4 haplotype (“APOE_genetic”) have extremely high explanatory signals in the KRONOSII data (solid lines) with the *VGF* signal actually exceeding *APOE* in KRONOSII. *VGF* is also high in the RUSH dataset (dotted lines), but not strong enough to overtake the main *APOE* signal. Interestingly, *RANBP3L* is also a MMI hit in both datasets, whereas our other hits only appear to have strong effects in KRONOSII. Finally, we tested *APOE* expression levels for MMI with DX. *APOE* expression is not a robust signal in either set. This matches with our finding that there is no relationship between *APOE* haplotypes and *APOE* expression when just plotting expression without considering MMI (Supplementary Fig. 7).

## Discussion

In this study we have demonstrated the high utility of the BN-driven computational systems biology approach for studying LOAD. The principal advantages of the BN modeling outlined in Introduction above (removal of spurious relationships; mixed data types handling; no linearity or normality assumptions; combination of both network visualization/interpretation and classification, at high levels of granularity) proved to be instrumental in our analysis. In this, we concur with the recent LOAD literature^24–26^. We believe the BNs to be a robust, flexible and fitting tool, around which further multiscale network modeling in the LOAD context should be centered. The main impediment to a wider BN modeling application is the limited scalability due to the high computational complexity; however, this is now less of an issue due to the increasingly better hardware, and continuing progress in both algorithmic development and software implementation^11,24,27^. Notably, it is precisely this high computational complexity that enables filtering out spurious correlations and transitive dependencies, inherent to the datasets with complex correlation structure, which is the case with LOAD. This distinguishes BN modeling from the more straightforward (i.e., relying on pairwise correlations) network-centric approaches currently predominant in the LOAD computational analyses, such as protein–protein interaction (PPI) networks, gene co-expression and regulatory networks, and multi-scale networks^28–31^. Concurrently, BN modeling, although a foundational machine learning (ML) method, emphasizes intrinsic interpretability, which contrasts favorably with the majority of the high predictive performance-oriented ML/DL (deep learning) methods that have recently been gaining traction in the LOAD space^31–33^, as DL explainability is largely limited to the *ex post facto* feature attribution and/or broad DL layer-level interpretation. Overall, we see BNs as a “happy medium” methodology, complementing both conventional network-centric approaches and emerging DL techniques in a LOAD computational analysis toolkit.

Our most robust hit was *VGF*. High levels of VGF are protective against the development of Alzheimer's disease generally, and on an *APOE* E4 risk background there is approximately a 50% reduction in the proportion of individuals with LOAD when there are high levels of VGF (see Fig. 4, yellow box). This hit was first implicated in LOAD using SELDI-TOF–MS to find novel biomarkers in patient CSF^34^. This initial CSF result has now been validated in the Alzheimer's Disease Neuroimaging Initiative (ADNI)^35^. Differences in *VGF* profiles have also been mapped in brain tissues^24,36^ further implicating this hit. VGF (nonacroymic) is a granin-like neuropeptide precursor protein which is processed by prohormone convertases^37^. Precursor cleavage results in several downstream peptides with various functions including inflammation^38^, pain^38^, reproduction^39^, energy metabolism/feeding^40^, and circadian rhythms^41^. Central to all these functions is the crucial role that VGF plays in the secretory pathway along with other members of the granin family^42^. The secretory pathway has long been implicated in Alzheimer's disease pathogenesis with the first findings coming from studies of EOAD, where the Swedish mutation results in differences in Amyloid precursor protein (APP) sorting^43^. *APOE* pathogenesis in LOAD also involves pathway regulation; however, in this case APP pathogenesis is likely caused by faster endocytosis through *APOE* E4 binding of the LRP1 receptor^44^. It remains to be seen whether VGF may block this process directly, or whether the protection from overexpressed VGF is merely due to its growth factor properties.

For all the other hits we mapped, increases in expression caused increases in risk for LOAD development. SPECC1L colocalizes with tubulin and actin. Deficiencies in SPECC1L protein expression can lead to deficits in vertebrate facial morphogenesis^45^. SPECC1L is thought to be a regulator of adherens junction stability and remodeling of the actin cytoskeleton^46^. Loss of SPECC1L increases staining of adherens junctions^47^, and therefore, increased expression of *SPECC1L*, as seen in our study, may be involved in risk via breakdowns in blood brain barrier (BBB) integrity. *APOE* also acts on BBB integrity. Specifically, the E4 containing forms of *apoE* increase age-dependent breakdowns in the blood brain barrier in mouse models ^48^.

HLA-DRA is a member of HLA class II set of proteins, which sit on the surface of antigen presenting cells and act in pathogen recognition. Upregulation of HLA class II proteins is a marker of activated microglia in LOAD^49^ and AD patients have a higher load of CD4^+^HLA-DR^+^ and CD8^+^HLA-DR^+^ lymphocytes^50^. In large, multisite genome-wide association screens variants near the *HLA-DRB5-DRB1* cluster just downstream of *HLA-DRA* are consistently found to be associated with LOAD risk^51,52^. HLA is known to be involved in immune inflammatory response^53^, as well as CNS plasticity and signal transmission for the HLA MHC class I proteins^54^. In the context of *APOE* genetic risk, prior work has focused on class I proteins, showing protective effects of *HLA-DRB* haplotypes^55^; however, this is counter to the GWAS results, where *HLA-DRB* and *HLA-DQA/B* are risk factors^51,52^. Further work has suggested that HLA-antigen incongruence resulting in persistent immune activation can lead to risk for AD^56^, which is in concordance with our finding of *HLA-DRB* activation. It remains to be seen how *APOE* haplotypes, *HLA-DRB* haplotypes and HLA-DRA expression activation interact.

RANBP3L is a nuclear pore protein which enables nuclear protein translocation through the nuclear pore complex (NPC). RANBP3L specifically acts on BMP-specific SMAD 1/5/8 proteins and terminates BMP signaling by blocking nuclear import^57^. Reductions in SMAD signaling have been linked to AD pathogenesis in both neurons and glia ^58,59^. Prior work on RANs in AD showed reduced expression of *RAN*, *RANBP1*, *RANBP2*, *RANBP5*, *RANBP9* and *RANBP10* in AD tissues, proposing that the toxic forms of *APP* knocked down RANs and thus reduced import into the nucleus^60^. RANBP3L was not measured in that screen. In our series there are also seen statistically significant reductions in *RAN*, *RANBP1*, *RANBP6*, and *RANBP9*, which is consistent with the prior report^5^; however, none of these downregulated hits were crucial to the *APOE* effect. From our studies the crucial effect is an upregulation in *RANBP3L* blocking SMAD signaling by blocking nuclear import. It remains to be seen how *APOE* E4 acts in concert with *RANBP3L*.

Our results complement other recent studies exploring multiscale network analyses in the LOAD context. Guo et al.^61^ used multiscale network analyses applied to large-scale human postmortem brain transcriptomic data of LOAD from two cohorts to dissect the interplay of APOE, sex, and LDL receptor related protein 10 (LRP10) as a key LOAD driver. Pan et al.^62^ converged on a VGF/DUSP4 (Dual-Specificity Protein Phosphatase 4) network. Neff et al.^63^ identified, via multiscale network analysis, LOAD subtype-specific drivers such as GABRB2, LRP10, MSN, PLP1, and ATP6V1A. These and other recent studies illustrate the growing power and the emerging promise of multi-scale and integrative network approaches to reveal and subtype novel LOAD biology^64–68^, and underline the ability of such approaches to dissect the interactions between APOE, VGF and emerging novel LOAD targets.

A notable limitation of our BN approach is the unbounded nature of the conventional BN scoring criteria (MDL/BIC, AIC). MDL is approximately linearly proportional to the sample size, and is dependent on the variables’ complement and the global network context, thus making direct inter-cohort comparisons difficult, with edge strengths not being commeasurable across the BNs. This incongruity contributed to our difficulties in reconciling KRONOS and RUSH cohort results. An additional limitation is the absence of the principled, rigorous power analysis framework for BN modeling in the genetic epidemiology context. In the future, we plan to explore alternative, commensurate network scoring criteria and edge strength measures.

In conclusion, our study seeks to define the role of *APOE* risk haplotypes on the downstream effects of expression being agnostic to genomic location or biological hypothesis. We have used MI and BN modeling to define ***novel*** hits which can either increase risk or offer protection against developing LOAD in the context of risk conferred by the *APOE* E4 haplotype. This study extends prior work in that we are sampling all expression profiles, rather than just looking at genomic locations closest to *APOE*, which is a common technique in GWAS follow-up or *cis* expression profiling. We are additionally performing network analysis in the context of risk genotypes, which is also not widely done as most network profiling focusses on continuous variable data only (i.e. expression). Network analysis, and specifically BN modeling, offer distinct advantages for the reasons stated above. Finally, we have demonstrated that power is a crucial component to these screens, with reproducibility only seen where there are enough alleles/haplotypes to capture effects. Further work will involve an understanding of additional genetic risk hits beyond *APOE* as well as compound modeling, where power permits. This will be facilitated by a commensurate BN scoring criteria, allowing for robust comparisons, transfer learning and power estimation frameworks.

## Conclusion

Taking a combined approach to network building including the *APOE* E4 locus, our findings nominate *VGF*, *SPECC1L*, *HLA-DRA* and *RANBP3L* as novel expression loci involved in the pathogenesis of LOAD. Of particular interest, high levels of VGF are protective on an *APOE* E4 background.

### Supplementary Information

Supplementary Figures.

## Data Availability

The datasets generated during and/or analyzed during the current study are available in the Laboratory of Functional Neurogenomics website (https://xzmxbgsv808roffneicreq.on.drv.tw/www.lfun/LFUN/LFUN/INDEX.html). Relevant code and software are available directly from the authors, or as part of the BNOmics package, at https://bitbucket.org/77D/bnomics.
